# Stromal CD10 expression in gastric adenocarcinoma

**DOI:** 10.25122/jml-2021-0244

**Published:** 2022-05

**Authors:** Sara Jalal Aziz, Jalal Ali Jalal, Kalthuma Saleh Hamadameen

**Affiliations:** 1. Department of Histopathology, Rizgary Teaching Hospital, Erbil, Iraq; 2. Department of Basic Sciences/Pathology, College of Medicine, Hawler Medical University, Erbil, Iraq

**Keywords:** gastric adenocarcinoma, stromal CD10, immunohistochemistry, stromal myofibroblasts

## Abstract

Gastric adenocarcinoma is a malignant neoplasm of the gastric mucosa composed of neoplastic cells and supporting stroma as with any neoplasm. Stromal myofibroblasts have an essential role in creating the tumor-promoting environment. They express certain substances, such as CD10. In this study, stromal CD10 expression was investigated by immunohistochemistry in gastric carcinoma, and its association with specific clinicopathological parameters was analyzed. Formalin-fixed paraffin-embedded blocks of 80 gastric adenocarcinoma cases were collected retrospectively in a private laboratory of the Rizgary Teaching Hospital for 2 years (January 2018–January 2020). Finally, the immunohistochemical study of CD10 expression in stromal cells was performed. According to the results, stromal CD10 immunoreactivity was detected in 15% of the cases. Furthermore, a statistically significant correlation was observed between stromal CD10 and the tumor type (P=0.015). However, no statistically significant relationship was identified between stromal CD10 expression and patients' age, gender, lymphovascular invasion, lymph node status, and tumor stage and grade. The results suggest a statistically significant positive correlation between stromal CD10 expression and tumor type.

## INTRODUCTION

Gastric cancer (GC) is the fifth most common cancer and the third leading cause of cancer-related deaths (high-rate mortality) in the world [1, 2]. A survey in Iraq revealed that GC is the seventh most common cancer [3]. Many patients have advanced or metastatic disease at presentation, and their prognosis is relatively poor [4]. GC has heterogeneous biological behavior resulting in differing prognoses independent of the clinical stage [5].

Carcinogenesis is a multistep process involving genetic mutations of neoplastic cells and the development of supportive stroma [6]. Despite extensive studies on cancer cells, the research advances have demonstrated that cancer progression is basically dependent on individual biological behaviors, being controlled via the interaction between cancer cells and the tumor microenvironment (TME) [7]. Many researchers have identified the pivotal function of TME in GC progression [8–10].

TME, which is heterogeneous in nature, is responsible for the growth and expansion of cancer cells composed of an extracellular matrix (ECM) and various cell types, including fibroblasts, immune cells, and vascular endothelial cells [11, 12]. These are stromal cells that release different molecules to directly activate the growth signaling in cancer cells or remodel surrounding areas to help tumors [11].

Stromal myofibroblasts, called cancer-associated fibroblasts (CAF), include both tumor-promoting and tumor-restraining populations [11]. They have several origins, such as marrow-derived mesenchymal stem cells or stromal fibroblasts' transformation by tumor-derived cytokines (*e.g*., transforming growth factor-β (TGF-β)) [13]. Due to their heterogeneity, CAFs are not defined by a single marker but rather as a cell that expresses ECM molecules and matrix metalloproteinases (MMPs) [14]. CD10-positive stromal cells belong to the myofibroblast group [15].

Evidence shows that myofibroblasts have a significant role in tumor growth, invasion, and metastasis [6, 16]. CAFs express some MMPs that have been reported to promote epithelial invasion [17]. These MMPs contribute to cancer development by releasing bioactive molecules that prevent apoptosis and promote invasion and metastasis, degradation of ECM components, induction of angiogenesis, and modulation of the immune system [18, 19].

CD10 is a cell surface type II zinc-dependent metalloprotease identical to neutral endopeptidase; it has structural similarity to MMPs and is one of the intestinal markers in patients with early gastric cancer [20, 21]. Its DNA sequences are located on human chromosome 3, at 3q21-27 [22]. Moreover, it is considered a marker for germinal center cells of the normal lymphoid tissue and their derivative follicular lymphomas [23]. It is also highly expressed in kidney and lung tissues and might be found in the choroid plexus, placenta, gonads, adrenal cortex, and small intestine [21]. The stroma of various malignancies contains CD10 positive cells, *e.g*., gastric, lung, breast, and prostate carcinomas [24–27].

The recognition of stromal CD10 in gastric cancer might predict invasive and metastatic potency and assist in the discovery of targeted therapy.

This study aimed to demonstrate stromal CD10 immunoreactivity in gastric adenocarcinoma and investigate its association with some clinicopathological parameters, including the patient's age, gender, tumor type, lymphovascular invasion, and lymph node status as well as tumor grade and stage.

## MATERIAL AND METHODS

Eighty formalin-fixed paraffin-embedded blocks of partial or total gastrectomy specimens performed for gastric adenocarcinoma were collected retrospectively from the Rizgary hospital and a private pathology laboratory in Erbil city (Kurdistan region of Iraq) for 2 years, from January 2018 to January 2020.

Two sections were prepared from each block, one stained with H&E to assess the amount of stroma and the other to evaluate stromal CD10 expression using immunohistochemistry.

Cases were classified based on their type to intestinal and diffuse types. Their histological grades were defined as well-moderately and poorly differentiated. The pathological staging was performed according to the 8^th^ edition of the American Joint Committee on cancer by grouping the various TNM components [28].

### Immunohistochemical Method

According to Dako's recommendations, sections were stained with CD10 immuno-stain using labeled and enhanced polymer systems (Dako EnVision™ Flex). In the Dako autostainer PT link, staining steps and incubation times are pre-programmed. The sections with 4 µm thick were mounted on salinized slides and put in an autostainer (in which a substrate buffer, blocking reagent for endogenous peroxidase, monoclonal anti-human CD10 as a primary antibody, mouse linker as a secondary reagent, EnVision/HRP as a labeled polymer, chromogen, a counterstain as hematoxylin, and distilled water were applied on all slides). After removing the autostainer slides, they were put in graded ethanols of 70%, 100%, and 100% for 2 minutes each; then, they were put for 2 minutes in xylene. Lastly, mounting was performed using Canada Balsam. Scoring of stromal CD10 immunoreactivity was done and reviewed by two expert pathologists using light microscopy. A section from the reactive lymph node was used as a positive control for CD10 immunoreactivity, while the negative control was checked by omitting the primary antibody.

### Scoring System

The cutoff value of antibody reactivity was 10%. CD10 immunoreactivity in more than 10% of stromal cells was regarded as positive, while any positivity of less than 10% of stromal cells was regarded as negative [24, 29, 30].

### Statistical Analysis

We depended on SPSS program version 23 for statistical analysis; the significance level was set (p≤0.05). The chi-square test was used to study the association between CD10 expression and clinicopathological parameters.

## RESULTS

Out of 80 cases, 44 (55%) were males, and 36 (45%) were females. The male-to-female ratio was 1.2:1. Their age ranged from 26 to 90 years, with a mean age of 61.21 years. Furthermore, about 80% of the patients were above 50 years. The data of other clinicopathological features of all cases are summarized in Table 1.

**Table 1:** The demographic and clinicopathological features of sampled cases.

Variables	Categories	Number	Percent
**Gender**	**Male**	44	55
	**Female**	36	45
**Age groups**	**≤50 years**	16	20
	**>50 years**	64	80
**Tumor type**	**Intestinal type**	48	60
	**Diffuse type**	32	40
**Tumor grade**	**Well-moderately differentiated**	33	41.3
	**Poorly differentiated**	47	58.7
**Lymphovascular invasion**	**Positive**	64	80
	**Negative**	16	20
**Nodal status**	**Positive**	69	86.3
	**Negative**	11	13.8
**Tumor stage**	**1–2**	24	30
	**3–4**	56	70
**Stromal CD10 expression**	**Positive**	12	15
	**Negative**	68	85
**Total**		80	100

Analysis of stromal CD10 immunoreactivity in all 80 samples showed that only 12 (15%) cases were positive for stromal CD10, and the remaining 68 (85%) were negative.

Among positive cases, 11 (91.6%) were intestinal type, and 1 (8.3%) was a diffuse type; whereas, among negative cases, 37 (54.4%) were intestinal type and 31 (45.6%) were diffuse type. Stromal CD10 expression was significantly correlated with tumor type (p=0.015).

The tumor grades of 33 samples were well-moderately differentiated, and 47 were poorly differentiated. From each grade group, 6 cases were positive for stromal CD10.

Further, 64 cases had positive lymphovascular invasion, and 69 cases had positive lymph nodes. Out of 12 positive cases, 11 (approximately 92%) had lymphovascular invasion, and the same percentage had lymph node metastasis. However, most patients with positive lymphovascular invasion and lymph nodes had stromal cells negative for CD10.

The number of patients with tumor stage 1 or 2 was 24, while with stage 3 or 4 it was 56. Although most stromal CD10 positive cases (75%) were in stage 3 or 4 of the disease, they did not show stromal CD10 immunoreactivity.

There was no statistically significant association between stromal CD10 expression and tumor grade, lymphovascular invasion, lymph node status, and tumor stage. Association between stromal CD10 immunoreactivity and clinicopathological parameters is presented in Table 2.

**Table 2:** The correlation between stromal CD10 immunoreactivity and clinicopathological parameters.

Variables	Categories	Stromal CD10 expression		p-value
		Negative	Positive	
**Gender**	**Male**	38 (55.9%)	6 (50%)	0.706
	**Female**	30 (44.1%)	6 (50%)	
**Age groups**	**≤50 years**	15 (22.1%)	1 (8.3%)	0.442
	**>50 years**	53 (77.9%)	11 (91.7%)	
**Tumor Type**	**Intestinal type**	37 (54.4%)	11 (91.6%)	**0.015**
	**Diffuse type**	31 (45.6%)	1 (8.3%)	
**Tumor grade**	**Well-moderately differentiated**	27 (39.7%)	6 (50%)	0.539
	**Poorly differentiated**	41 (60.3%)	6 (50%)	
**Lymphovascular invasion**	**Positive**	53 (77.9%)	11 (91.7%)	0.442
	**Negative**	15 (22.1%)	1 (8.3%)	
**Nodal status**	**Positive**	58 (85.3%)	11 (91.7%)	0.479
	**Negative**	10 (14.7%)	1 (8.3%)	
**Tumor stage**	**1–2**	21 (30.9%)	3 (25%)	0.486
	**3–4**	47 (69.1%)	9 (75%)	
**Total**		68 (100%)	12 (100%)	

**Figure 1:**
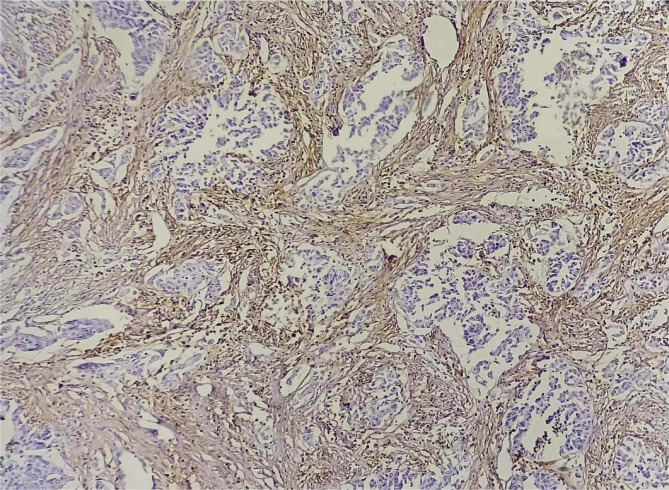
Positive stromal CD10 (IHC X100).

**Figure 2:**
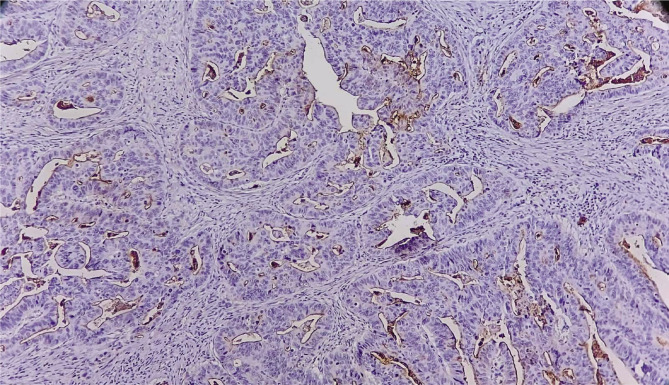
Negative stromal cells for CD10; Positive internal control can be noted on the surface of neoplastic glands (IHC X100).

Representative examples of a stromal CD10 positive and negative cases are shown in Figures 1 and 2, respectively.

## DISCUSSION

CD10 is a well-known metalloproteinase expressed by various cell types [21]. Their release from stromal myofibroblasts in malignant tumors enhances the growth of cancer cells [17].

Among the 80 patients, only 12 (15%) showed stromal CD10 immunoreactivity. This result was similar to that found by Wen-Bin Huang et al. [24], in which stromal CD10 positivity was identified in 19% of the sampled cases.

The present study demonstrated a statistically significant correlation between stromal CD10 and gastric adenocarcinoma type. Similar results were found by Wen-Bin Huang et al. [24], who studied stromal CD10 expression in 116 specimens of gastric cancer. He found a statistically significant association between such immunoreactivity and tumor type, as he observed that most stromal CD10 positive tumors were of intestinal type. Intestinal-type gastric adenocarcinoma arises from *Helicobacter pylori*-associated chronic gastritis that induces intestinal metaplasia. It undergoes dysplasia followed by carcinoma *in situ* and invasive carcinoma, and chronic inflammation recruits bone marrow-derived myofibroblasts positive for CD10 [13].

The correlation analysis between tumor grade and stromal C10 immunoreactivity did not show any significant result since, among 47 cases with high-grade histology, only 6 were stromal CD10 positive, and the remainder were negative; a similar result was found by Sravan et al. [29].

Compatible results about such association with lymphovascular invasion and lymph node status were found by Jafarian et al. [31]. Although a majority (about 92%) of stromal CD10 positive samples had the lymphovascular invasion, and the same percentage had positive lymph nodes, they were not significant statistically because 77% and 85% of stromal CD10 negative cases had lymphovascular invasion and lymph nodes, respectively. In contrast to the present study, Wen-Bin Huang et al. [24] demonstrated a significant correlation of stromal CD10 positivity with lymphovascular invasion and nodal metastasis. Additionally, a further study examining 78 cases of gastric lesions found a significant relation between CD10 and lymphoid node metastasis [32].

In the current research, a correlation was observed between stromal CD10 positivity and tumor stage, as 75% of positive cases were either stage II or IV; however, this relation was not statistically significant. This finding was in line with two similar studies done by Sravan et al. [29] and Wen-Bin Huang et al. [24]. In contrast, in Jafarian et al. study, the evidence demonstrated a relation between stromal CD 10 expression and tumor stage (p=0.01) [31].

Although the majority of positive cases were aged above 50 years, there was no significant correlation, similar to the results found by Jafarian et al. [31].

Earlier, Pan et al. [33] showed that peptide prodrugs improved the efficacy of cytotoxic drugs; however, they were cleavable by peptidases, including CD10 present in the tumor environment, according to which blockade of CD10 increased the therapeutic index of such drugs. New cancer therapy to enhance the effect of already present cytotoxic drugs and, more interestingly, medications to prevent gastric cancer in high-risk patients can be invented depending on the present research and future studies on the molecular basis of stromal–cancer interaction.

The discrepancy between the findings of this research and those of previous studies is probably due to several factors, including sample size, fixation duration, antigen retrieval method, antibody type, and scoring system. In the present study, a correlation of stromal CD10 expression with lymphovascular invasion and lymph node metastasis at a higher stage was observed; however, these correlations were not significant statistically.

## CONCLUSION

The correlation of stromal CD10 expression with some clinicopathological parameters, such as age, gender, lymphovascular invasion, lymph node status, as well as tumor grade and stage, appears to be helpful. In this study, stromal CD10 immunoreactivity was identified in 15% of gastric adenocarcinoma cases, which was significantly correlated with the tumor type; however, no statistically significant association was identified with other parameters.
